# Sorting Test, Tower Test, and BRIEF-SR do not predict school performance of healthy adolescents in preuniversity education

**DOI:** 10.3389/fpsyg.2014.00287

**Published:** 2014-04-08

**Authors:** Annemarie Boschloo, Lydia Krabbendam, Aukje Aben, Renate de Groot, Jelle Jolles

**Affiliations:** ^1^Department of Educational Neuroscience, Faculty of Psychology and Education, LEARN! Research Institute, VU University AmsterdamAmsterdam, Netherlands; ^2^Hogeschool iPaboAlkmaar, Netherlands; ^3^Het Bouwens van der BoijecollegePanningen, Netherlands; ^4^Centre for Learning Sciences and Technologies, Open Universiteit NederlandHeerlen, Netherlands

**Keywords:** neuropsychology, executive functions, adolescence, academic performance, education

## Abstract

Executive functions (EF) such as self-monitoring, planning, and organizing are known to develop through childhood and adolescence. They are of potential importance for learning and school performance. Earlier research into the relation between EF and school performance did not provide clear results possibly because confounding factors such as educational track, boy-girl differences, and parental education were not taken into account. The present study therefore investigated the relation between executive function tests and school performance in a highly controlled sample of 173 healthy adolescents aged 12–18. Only students in the pre-university educational track were used and the performance of boys was compared to that of girls. Results showed that there was no relation between the report marks obtained and the performance on executive function tests, notably the Sorting Test and the Tower Test of the Delis-Kaplan Executive Functions System (D-KEFS). Likewise, no relation was found between the report marks and the scores on the Behavior Rating Inventory of Executive Function—Self-Report Version (BRIEF-SR) after these were controlled for grade, sex, and level of parental education. The findings indicate that executive functioning as measured with widely used instruments such as the BRIEF-SR does not predict school performance of adolescents in preuniversity education any better than a student's grade, sex, and level of parental education.

## Introduction

At school, adolescents often get complex assignments and have to do homework for various courses simultaneously. In addition, they have to decide which combinations of courses to follow, which in turn may affect their possibilities for higher education and future careers. Therefore, the adolescent student needs to develop higher cognitive skills, such as self-monitoring, planning and organizing, in order to perform well. It is unclear, however, whether the development of these functions also predicts adolescents' school performance. Insight into the cognitive predictors of school performance is relevant for school (neuro) psychologists and other professionals who work with adolescents. They often have to estimate how scores on intelligence tests and neuropsychological tests are related to task performance in adolescents' daily life, for example to performance at school.

The neuropsychological measures often used for estimating performance in daily life are executive function tests (Gioia and Isquith, 2004; Chan et al., 2008). Executive functions are the functions necessary for goal-directed behavior (e.g., Best and Miller, 2010). A wide range of executive functions have been described in literature, such as inhibition, updating working memory, shifting, planning, organization skills, attentional control, and self-control (Alvarez and Emory, 2006; Best and Miller, 2010; Hofmann et al., 2012). However, concerns have been raised about the *ecological validity* of executive function tests; that is, how well they predict performance in daily life (Gioia and Isquith, 2004; Chan et al., 2008; Olson et al., 2013). Previous studies that related executive function tests to school performance in adolescents found mixed results (e.g., Gioia and Isquith, 2004; St Clair-Thompson and Gathercole, 2006; Chan et al., 2008; Latzman et al., 2010; Best et al., 2011), which we will address in depth below. These mixed results may have been caused by a lack of control for important confounders (Willoughby et al., 2012). Therefore, the present study set out to investigate whether performance on executive function tests is related to school performance in a highly controlled sample of adolescents.

Neuroscientific research associates executive functions with the functioning of neural networks between several brain areas including, but not restricted to, the prefrontal brain areas (Alvarez and Emory, 2006). These brain areas develop during childhood through adolescence until early adulthood (Gogtay et al., 2004; Giedd, 2008). Neuropsychological studies have confirmed that executive functions develop during this time period, with some functions becoming fully developed earlier than others (Anderson, 2002; Best and Miller, 2010). Considering the nature of executive functions, and the fact that they are still developing during adolescence, it is likely that adolescents' school performance is related to the degree of maturation of relevant executive functions.

As we mentioned earlier, studies investigating the relation between executive functions and school performance have reported mixed results. These studies can be classified by the outcome measures they used to assess school performance. School performance can be measured with various outcome measures, such as report marks or performance on standardized tests. Of these two measures of school performance, report marks have the highest ecological validity, since they are relevant for students' daily lives. Decisions on passing or failing a course or grade are made based on report marks.

Only a few studies have investigated the relation between executive functions and report marks. Most of these studies were conducted with young adolescents, aged 12–13 (Veenman et al., 2005; Checa et al., 2008; Checa and Rueda, 2011). Results showed that executive functions such as executive attention (Checa et al., 2008), and metacognitive (executive) skills (Veenman et al., 2005), partially predicted report marks for mathematics. Attention and effortful control were found to be related not only to performance in mathematics, but also to the average report mark of all subjects at the end of the academic year (Checa and Rueda, 2011). In primary school children in third grade, executive function tests such as the Trail Making Test and the Tower of Hanoi did not relate to report marks. However, performance on a classroom-based planning task and teacher reports on children's time management skills were related to report marks (Cohen et al., 1995).

Most studies that have investigated the relation between executive functions and school performance in adolescents did not consider report marks, but looked at the outcomes of standardized performance tests. Standardized tests are equal for all students, and scores are not dependent on a student's school, class or teacher, as is the case with report marks. Studies on the relation between performance on standardized tests and executive functions showed that girls' performance on mathematics in adolescence and early adulthood was predicted by executive functioning measured in childhood, and especially by the score obtained on the Rey Osterrieth Complex Figure (Miller and Hinshaw, 2010; Miller et al., 2012). Furthermore, in a cross-sectional study, three complex executive function measures from the Cognitive Assessment System were related to school performance on reading and mathematics in children and adolescents aged 5–17 (Best et al., 2011). Other studies found that not all executive function tests contributed equally to various academic skills. Results from one study on adolescent boys aged 11–16 (Latzman et al., 2010) showed that: the Delis-Kaplan Executive Functions System (D-KEFS) composite score for conceptual flexibility was related to performance in reading and science; the monitoring composite was related to reading and social studies; and inhibition was related to mathematics and science. Another study on adolescents aged 11–12 (St Clair-Thompson and Gathercole, 2006) reported that: updating was related to performance in English and mathematics; inhibition was related to English, mathematics, and science; and that shifting was not related to school performance.

As these studies show, performance on some executive function tests appears to be related to school performance. However, the study results vary when it comes to determining which specific executive functions are related to different school subjects, and they are inconclusive about the exact extent of the relationships. Studies that report high correlations between executive functions and school performance (between 0.30 and 0.50 or higher), often used a sample that had diverse socioeconomic backgrounds, or they did not control for sex or intelligence (e.g., St Clair-Thompson and Gathercole, 2006; Best et al., 2011). Studies that did control for these factors generally found lower correlations (around 0.10–0.25) (Latzman et al., 2010; Miller and Hinshaw, 2010). Clearly, in order to investigate the association between executive functions and school performance, it is crucial to carefully control for confounders. In addition, research in younger children shows that the relationship between executive functions and school performance is confounded by unmeasured variables that are constant over time, such as household or care-giver characteristics (Willoughby et al., 2012).

As it is impossible to measure all potential confounders that may affect executive functions, the current study used a homogeneous sample. In that way, we were able to control for many known and unknown variables. Our sample consisted of students in the preuniversity educational track, which is the most advanced track of the Dutch secondary school system; the top 20% of all students in Dutch secondary education are in this track (Ministry of Education, Culture and Science, 2009). Therefore, all our participants were high-performing students. Moreover, we selected students who had never repeated or skipped a grade in school. Studies show that students who have repeated or skipped a grade have different profiles with regard to a range of school related variables compared to students with a regular educational career (Jimerson, 2001; Steenbergen-Hu and Moon, 2011). In addition, given that the former are a year older or younger than their classmates, they are most probably at a different stage of biological and psychological development. Finally, we reduced the effects of medical factors that may influence the relation between executive functions and school performance, such as past brain trauma, a developmental disorder, or medication use, by including only healthy, normally developing adolescents. Because of these selection criteria, our sample was homogeneous with regard to both ability level and developmental history.

The current study investigated one possible confounder in particular, namely sex. Sex is well known for its influence on school performance, as girls and boys excel at different subjects (Machin, 2005; Van Langen et al., 2006; Clark et al., 2008; Driessen and Van Langen, 2010). There is also growing evidence to support the conclusion that the neuropsychological performance of boys and girls differs in the school setting (Martens et al., 2011; Dekker et al., 2013a). In addition, a recent study has reported differences in executive functioning related to school performance in adolescents of different sex (Coenen et al., 2011). Other studies have shown that girls perform better in the school setting because they are better at self-control and self-discipline (Downey et al., 2005; Duckworth and Seligman, 2006; Hyde et al., 2007; Steinmayr and Spinath, 2008), which is interesting as there are indications that executive functions subserve self-control and self-discipline (Hofmann et al., 2012). Finally, evidence is accumulating that boys and girls differ in brain maturation, especially during an extended period in adolescence, with boys lagging behind (Giedd, 2008; Lenroot and Giedd, 2010). This suggests that biological factors pertaining to brain development may underly the complex relation between executive functions and school performance. Other factors may influence the relation between sex and school performance as well. For example, boys are more likely to show work-avoidant motivational strategies than girls in secondary education (Dekker et al., 2013b). It may thus be that a relation between executive function and school performance is visible in girls, but not in boys. This hypothesis is addressed in the present study.

In sum, the main aim of the current study was to investigate the respective relations between three different measures of executive functions and school performance, while keeping close control of confounders. In addition, we investigated whether these relations were moderated by sex. We investigated a homogeneous sample of 173 healthy adolescents, all secondary school students in the pre-university educational track. Two objective, performance-based neuropsychological tests were used to measure categorizing and shifting (Sorting Test from the D-KEFS) and planning skills (Tower Test from the D-KEFS). These tests are suitable for administration to adolescents, and measure executive functions that are still developing at this age range (Delis et al., 2001; Huizinga et al., 2006; Luciana et al., 2009). Furthermore, we also administered the Behavior Rating Inventory of Executive Function—Self-Report Version (BRIEF-SR) (Guy et al., 2004). This questionnaire has been developed to measure a wide range of executive functions based on their appearance in real-world behavior. Therefore, the BRIEF-SR has been claimed to be a more ecologically valid measure of executive functions than objective neuropsychological tests (Gioia and Isquith, 2004; Guy et al., 2004; Olson et al., 2013). We used report marks to measure school performance, since these are most relevant to adolescents themselves. School performance was measured with the end-of-term report marks for Dutch (the native language), English as a foreign language, and mathematics. Based upon the assumption that the BRIEF-SR is more ecologically valid than objective executive function tests, we hypothesized that the BRIEF-SR would predict report marks better than the objective tests. In addition, we hypothesized that the relation between executive functions and school performance would be different for boys and girls.

## Methods

### Participants

Participants came from seven secondary schools in the south of the Netherlands. They were in grade 7, 9, or 11 of the pre-university educational track. This is the most advanced track in Dutch secondary education; the top 20% of all students in Dutch secondary education are in this track (Ministry of Education, Culture and Science, 2009). Participants had not repeated or skipped a grade. Furthermore, participants had the Dutch nationality, had no learning disorders, psychiatric disorders or developmental disorders, did not use medication that influences cognitive functions and did not have a history of brain damage with a loss of consciousness of more than 30 min. These criteria were measured with a questionnaire that was completed by the parents.

The participants themselves and the parents of under-aged participants had to give permission for participation. Participants received a monetary reward for participation. The Ethical Committee of the Faculty of Psychology of Maastricht University approved the research protocol.

### Measures

#### Executive functions

Objective measures of executive functions were acquired with the Sorting Test and the Tower Test from the Delis-Kaplan Executive Functions System (D-KEFS) (Delis et al., 2001). The Sorting Test is a card sorting test that aims to measure categorization skills and set shifting. No Dutch version existed; therefore we translated the words on the cards, and changed some words to make all original sorts possible. The free sorting condition was used. Outcome measure of the Sorting Test was the number of confirmed correct sorts (range: 0–16). The Tower Test measures planning, and has a strong learning component due to the nature of the items. The raw total achievement score was used as outcome measure (range: 0–30).

As a subjective measure of executive functions, the Behavior Rating Inventory of Executive Function—Self Report Version (BRIEF-SR) (Guy et al., 2004) was used. The BRIEF-SR is an 80-item questionnaire, especially developed for adolescents, in which they have to indicate how often the described behaviors had been a problem in the past six months (never, sometimes or often). The items can be grouped into 8 scales that measure the following executive functions: Inhibit, Shift, Emotional Control, Monitor (together: the Behavioral Regulation Index, BRI), and Working Memory, Plan/Organize, Organization of Materials, and Task Completion (together: the Metacognition Index, MCI). A higher score on the MCI and the BRI indicates more problems with executive functioning. Following the official Dutch translation of the BRIEF Parent Version (by Smidts and Sergeant), the BRIEF-SR was translated into Dutch. Few items of the BRIEF-SR are different from those in the Parent Version. These were translated by a native English-Dutch bilingual psychologist, and reviewed by another psychologist. The internal consistency of this Dutch version of the BRIEF-SR was *r* = 0.89 for the BRI and *r* = 0.91 for the MCI.

#### Report marks

End of term report marks (ranging from 1.0 = very bad to 10.0 = outstanding) for Dutch, English, and mathematics were acquired through the schools' administration. Dutch, English, and mathematics are the first three main goals of secondary education in the Netherlands (Ministry of Education, Culture and Science, 2006) and are valid estimators of school performance (Reed et al., 2010). These report marks are the result of multiple smaller and larger tests and assessments (at least more than 4) that were administered during one school year. The tests and assessments were part of the teaching method or were developed by teachers themselves, and could consist of various assessment methods, e.g., paper-and-pencil tests, essays, presentations. Because the schools in the sample used different grading policies, each school's report marks were transformed into z-scores, based on the school's mean report mark and its standard deviation. In this way, the distribution of scores was similar for each school.

#### Demographics

Participants reported age and sex. Parents reported both parents' education level. Level of parental education (LPE) was defined as the highest education of the two. LPE was medium when the parents had junior vocational or junior general secondary education and high when they had senior vocational or academic education.

### Procedure

Adolescents were recruited through letters that were distributed at the seven schools by the researchers. All students were in grade 7, 9, or 11 at the start of the study. Because the study started at the end of a school year, 50.9% of the adolescents were tested in the new school year, and were therefore in grade 8, 10, or 12 when they participated. A trained psychologist administered tests and questionnaires in a quiet room at school. Administration took approximately 1.5 h. Adolescents participated during school time and therefore missed certain lessons.

### Analyses

All analyses were performed with SPSS Statistics 19.0 for Mac. First, to examine relations between all variables of interest, zero-order correlations were calculated. To investigate whether executive functions predicted report marks after correction for grade at the start of the study, sex, and LPE, separate multivariate GLM analyses (MANCOVA) were performed for each executive function score. Dependent variables were standardized report marks for Dutch, English, and mathematics. The following fixed factors and covariates were included: grade, sex, LPE (hereafter called demographic variables) and executive function score. After investigating main effects, we investigated interaction effects. To examine whether results were different for the different grades, we added the interaction term grade ^*^ executive function score. To investigate influence of sex, analyses were performed with inclusion of the interaction between sex and executive function score. Finally, we investigated a model with all executive function scores and all demographic variables to investigate their contribution together, and the same model without demographic variables to investigate the amount of variance predicted by executive function scores alone.

## Results

A total of 173 adolescents between 12.68 years and 18.05 years participated (age *M* = 15.22 years; *SD* = 1.66). Of those, 63.6% had highly educated parents, and the remainder had parents with a medium education level. Table 1 shows outcomes on executive function measures and school performance, per grade and sex. Sex differences were seen on the Tower Test to the advantage of boys. On the other executive function measures, no sex differences were found. On all school report marks, there were differences between grades and between sexes: students from lower grades had higher report marks than students from higher grades, and girls achieved higher report marks than boys.

**Table 1:** **Descriptive statistics of executive function measures and school performance, per grade and sex**.

	**Total**	**Grade 7**		**Grade 9**		**Grade 11**		**Grade effect**	**Sex effect**
		**Boys**	**Girls**	**Boys**	**Girls**	**Boys**	**Girls**	**Test statistic**	**Test statistic**
*N*	173	32	28	30	33	20	30	−	−
Age	15.22 (1.66)	13.26 (0.43)	13.39 (0.35)	15.31 (0.36)	15.34 (0.30)	17.40 (0.37)	17.38 (0.40)	*F* = 1615.31^**^	*F* = 0.70
LPE *(% high)*	63.6	62.5	57.1	73.3	60.6	55.0	70.0	−	−
Sorting test confirmed correct score^a^	10.75 (1.57)	10.47 (1.52)	10.32 (1.85)	10.73 (1.64)	11.03 (1.43)	10.55 (1.50)	11.30 (1.34)	*F* = 2.17	*F* = 1.28
Tower test total achievement score^a^	18.12 (3.43)	18.03 (3.13)	18.14 (3.16)	18.73 (3.49)	17.15 (2.79)	20.00 (4.81)	17.40 (3.10)	*F* = 0.53	*F* = 6.03^*^
BRIEF−SR BRI^a^	54.99 (9.17)	56.59 (9.65)	54.50 (8.57)	51.17 (6.87)	55.88 (9.72)	58.00 (7.00)	54.57 (11.04)	*F* = 1.10	*F* = 0.00^b^
BRIEF−SR MCI^a^	64.79 (11.93)	64.47 (10.53)	61.43 (11.30)	63.13 (9.53)	63.72 (12.93)	74.00 (12.49)	64.93 (13.06)	*F* = 4.12^*^	*F* = 3.62
Report mark Dutch	7.06 (0.83)	7.16 (0.91)	7.83 (0.70)	6.60 (0.53)	7.29 (0.66)	6.30 (0.57)	6.98 (0.75)		
Report mark Dutch standard score	0 (0.98)	0.04 (0.96)	0.99 (0.69)	−0.51 (0.66)	0.22 (0.87)	−0.92 (0.69)	−0.08 (0.92)	*F* = 21.88^**^	*F* = 45.16^**^
Report mark English	7.27 (1.15)	7.30 (0.98)	8.03 (1.11)	7.05 (0.96)	7.55 (0.95)	6.85 (0.82)	6.71 (1.47)		
Report mark English standard score	0 (0.98)	0.13 (0.82)	0.78 (0.84)	−0.18 (0.82)	0.19 (0.84)	−0.45 (0.68)	−0.60 (1.17)	*F* = 17.60^**^	*F* = 5.48^*^
Report mark mathematics	6.89 (1.11)	7.20 (1.14)	7.53 (0.83)	6.63 (0.92)	6.95 (0.95)	5.90 (0.91)	6.79 (1.25)		
Report mark mathematics standard score	0 (0.98)	0.26 (1.06)	0.60 (0.72)	−0.18 (0.76)	0.06 (0.84)	−0.91 (0.83)	−0.11 (1.09)	*F* = 13.70^**^	*F* = 9.79^**^

*Values are M (SD). A dash indicates that the effect was not tested. Skewness of standard scores of report marks remained within an acceptable range (between −1 and +1). LPE, level of parental education; BRIEF-SR, Behavior Rating Inventory of Executive Function—Self-Report Version; BRI, Behavioral Regulation Index; MCI, Metacognition Index*.

a *Raw score*.

b Significant interaction effect between grade and sex, F = 3.38^*^.

* p < 0.05,

** *p < 0.01*.

### Relation between executive functions and report marks

Table 2 shows correlations between executive function measures and report marks. The BRI and MCI of the BRIEF-SR were the only executive function measures that significantly correlated with report marks. The BRI correlated with Dutch scores only (*r* = −0.17), while the MCI correlated with report marks in Dutch, English, and mathematics (between *r* = −0.20 to *r* = −0.27, *p* < 0.05). These correlations indicate that the more problems with behavior regulation a student reported, the lower the score for Dutch. In addition, the more problems with metacognition a student reported, the lower the score for Dutch, English, and mathematics.

**Table 2:** **Zero-order correlations between background variables, executive function measures, and report marks**.

	**1**	**2**	**3**	**4**	**5**	**6**	**7**	**8**	**9**	**10**
1. Group	1									
2. Sex	0.11	1								
3. LPE	0.04	−0.02	1							
4. Sorting Test confirmed correct score	0.16^*^	0.10	−0.01	1						
5. Tower Test total achievement score	0.04	−0.18^*^	−0.02	0.15	1					
6. BRIEF−SR BRI	0.01	0.00	−0.23^**^	−0.02	−0.01	1				
7. BRIEF−SR MCI	0.18^*^	−0.12	−0.08	0.06	0.15^*^	0.65^**^	1			
8. Standard score Dutch	−0.37^**^	0.39^**^	0.08	0.01	0.00	−0.17^*^	−0.27^**^	1		
9. Standard score English	−0.40^**^	0.12	−0.03	−0.06	0.01	−0.09	−0.20^**^	0.57^**^	1	
10. Standard score mathematics	−0.35^**^	0.18^*^	0.12	−0.02	0.10	−0.08	−0.20^*^	0.46^**^	0.50^**^	1

*LPE, level of parental education; BRIEF-SR, Behavior Rating Inventory of Executive Function—Self-Report Version; BRI, Behavioral Regulation Index; MCI, Metacognition Index*.

* p < 0.05,

** *p < 0.01*.

### Sorting test

MANCOVA analyses showed no significant main effect of Sorting Test score on report marks, *F*_(3, 165)_ = 0.27, *p* = 0.847, partial eta squared = 0.01. Repeating the analyses with interaction effects also showed no significant interaction effect between Sorting Test score and grade, and Sorting Test score and sex on report marks, resp. *F*_(6, 326)_ = 1.07, *p* = 0.382, partial eta squared = 0.02, and *F*_(3, 162)_ = 0.58, *p* = 0.631, partial eta squared = 0.01.

### Tower test

MANCOVA analyses showed no significant main effect of Tower Test score on report marks, *F*_(3, 165)_ = 1.98, *p* = 0.119, partial eta squared = 0.04. Repeating the analyses with the interaction effects also showed no significant interaction between Tower Test score and grade, and Tower Test score and sex on report marks, resp. *F*_(6, 326)_ = 1.49, *p* = 0.181, partial eta squared = 0.03 and *F*_(3, 162)_ = 0.64, *p* = 0.588, partial eta squared = 0.01.

### BRIEF-SR BRI

MANCOVA analyses showed no significant main effect of BRI score on report marks, *F*_(3, 165)_ = 1.99, *p* = 0.117, partial eta squared = 0.04. Repeating the analyses with the interaction effects also showed no significant interaction between the score on the BRI and grade, and the BRI and sex on report marks, resp. *F*_(6, 326)_ = 0.60, *p* = 0.729, partial eta squared = 0.01, and *F*_(3, 162)_ = 1.99, *p* = 0.118, partial eta squared = 0.04.

### BRIEF-SR MCI

MANCOVA analyses showed no significant main effect of MCI score on report marks, *F*_(3, 165)_ = 2.12, *p* = 0.100, partial eta squared = 0.04. Repeating the analyses with the interaction effects also showed no significant interaction between the score on the MCI and grade, and the MCI and sex on report marks, resp. *F*_(6, 326)_ = 0.57, *p* = 0.751, partial eta squared = 0.01, and *F*_(3, 162)_ = 1.03, *p* = 0.382, partial eta squared = 0.02.

### Model with all executive function scores

MANCOVA analyses with all executive function scores in one model showed no significant effects of any of the executive function scores [Sorting Test: *F*_(3, 162)_ = 0.17, *p* = 0.918, partial eta squared = 0.00; Tower Test: *F*_(3, 162)_ = 2.36, *p* = 0.074, partial eta squared = 0.042; BRIEF-SR BRI: *F*_(3, 162)_ = 0.66, *p* = 0.577, partial eta squared = 0.01; BRIEF-SR MCI: *F*_(3, 162)_ = 1.34, *p* = 0.262, partial eta squared = 0.02]. Investigating the three demographic variables showed that grade and sex were significant [grade: *F*_(6, 326)_ = 8.35, *p* < 0.001, partial eta squared = 0.13; sex: *F*_(3, 162)_ = 16.12, *p* < 0.001, partial eta squared = 0.23], and LPE approached significance [*F*_(3, 162)_ = 2.54, *p* = 0.058, partial eta squared = 0.05]. In all analyses performed in this article, these three demographic variables were included and their effects were as described in this analysis. A model without demographic variables, but with all executive function scores, showed a significant effect for the BRIEF-SR MCI [*F*_(3, 166)_ = 4.21, *p* = 0.007, partial eta squared = 0.07]. This effect is smaller than the variance explained by demographic variables in the previous analysis.

## Discussion

The current study investigated whether executive functions predicted report marks in healthy adolescents aged 12–18 who were secondary school students in the pre-university educational track. Results showed that performance on the Sorting Test and the Tower Test did not predict report marks for Dutch, English, and mathematics. There was a zero-order correlation between scores on the BRIEF-SR and report marks (*r* = 0.17–0.27). Such correlations are often reported in studies on the relation of executive function tests to school performance (e.g., St Clair-Thompson and Gathercole, 2006; Best et al., 2011). However, after correcting for grade, sex, and LPE, the BRIEF-SR did not predict report marks anymore. Moreover, sex did not influence the relation between executive functions and report marks, since the sex^*^ executive function score interaction term was not significant in any of our models.

The magnitude of the correlation between the BRIEF-SR and report marks in the current study was comparable to that found in studies that controlled for intelligence (Latzman et al., 2010; Miller and Hinshaw, 2010). In the current study, all participants were in the pre-university educational track, the level at which the top 20% of Dutch students is studying (Ministry of Education, Culture and Science, 2009). By selecting this high-performing sample, we used a group that is relatively homogeneous with respect to potential and intellectual ability [estimated intelligence quotient (IQ) higher than 90; (Van den Bos et al., 2012)]. Students in the pre-university educational track are overrepresented in higher socioeconomic status (SES) groups and there is some evidence that these students are generally more mature with regard to neuropsychological development (Hackman and Farah, 2009). The advantage of our study is that it enabled some control over possible confounders related to SES, and over slow (neuro) psychological development due to lack of environmental support. The findings can be considered as strong for the upper segment of the educational system (pre-university education). On the other hand, our design has the drawback that the results cannot be generalized to all adolescents and that we were not able to take IQ-scores into account. Forthcoming studies will address executive functions in relation to boy-girl difference in students in the “vocational” educational track; given the fact that this group is characterized by broader variance in SES, intelligence, learning motivation, and study results, findings are anticipated to be different than reported in the present paper. This would be relevant in terms of applicability of the findings in educational practice, notably in councelling of students and their parents and teachers with regard of the development of executive functions.

The fact that the BRIEF-SR did relate to report marks, while the objective neuropsychological tests (Sorting Test and Tower Test) did not, may illustrate the higher ecological validity of the BRIEF-SR. Yet, controlling for grade, sex, and LPE removed the relation between the BRIEF-SR and report marks. Thus, if one knows whether a student in a preuniversity educational track is a boy or a girl, which grade the student is in, and the educational level of the parents, one can predict report marks as well as with the score on the BRIEF-SR. This indicates that the BRIEF-SR measured aspects of performance in school that could be explained by grade, sex, and LPE.

Why could the executive function tests in the present study not predict school performance any better than grade, sex, and LPE? One explanation has to do with the fact that our study used a sample which was homogeneous with respect to educational track: only students from pre-university level were investigated, i.e., 20% of all students in secondary education. This choice will have reduced the influence of the background variables on the relation between executive functions and school performance. Our findings are in line with those of Willoughby et al. (2012), a study that resembles the present study by taking into account more than only measured confounders. Moreover, other studies that did not find any effects between executive functions and school performance may have remained unpublished. Another explanation is that students who are elected for the preuniversity track in secondary education are more mature in executive functioning in comparison to students in other educational tracks. This is of importance since—usually—, primary schools advise each student which educational level in secondary education is appropriate for them. This advice takes into account not only cognitive performance, but also expected development, motivation for school, and study approach (Driessen, 2005). It could be that, unknowingly, executive functions are taken into account as well. Students with good executive skills would then be advised to go to preuniversity education, while students with poorer executive skills would be advised to go to general secondary education or prevocational education. Future research should investigate the relation between executive functions and school performance in general secondary education and prevocational education as well. Possibly, effects will be found at these other educational levels.

However, also within preuniversity education, students themselves report that they differ with respect to their executive function skills (Coenen et al., 2011). Moreover, sex differences in self-control, which is closely linked to executive functions, appear to contribute to sex differences in school performance (Downey et al., 2005; Duckworth and Seligman, 2006; Hyde et al., 2007; Steinmayr and Spinath, 2008). This may indicate that the executive function tests used in this study were not sensitive enough to measure differences in high-performing healthy adolescents. Executive function tests used in clinical practice are often not sensitive enough to distinguish executive function difficulties in clinical groups (Chan et al., 2008), let alone in healthy subjects. Since each executive function test also measures other (not executive) functions, so called task-impurity, this may trouble the accurate measurement of executive functions (Miyake and Friedman, 2012). To accurately measure differences in executive functions between healthy high-performing adolescents, other tests, a combination of tests, or statistical methods such as the latent variable approach may be needed (Miyake and Friedman, 2012).

A strong point of the current study is that it used report marks to estimate school performance. Most studies measure school performance with standardized tests. An advantage of standardized tests is that these tests are similar for all participants in the study (OECD, 2007). A disadvantage of standardized tests is the lack of ecological validity, because standardized tests are not the outcomes on which students are being assessed in school (Cohen et al., 1995; Wolfson and Carskadon, 2003). Students are reliant on report marks for their school success, as report marks indicate whether a student may pass to the next grade or enter a certain school or educational track. Report marks may also have higher reliability than standardized tests because they involve multiple measurements and more closely measure learning that takes place at school (Wolfson and Carskadon, 2003). Thus, report marks may give a better estimation of real life outcomes than standardized tests.

The present study shows that school performance in healthy, high-performing adolescents could not be predicted by scores on the Sorting Test, Tower Test, and BRIEF-SR. It raises the question whether and to what extent school performance in this sample depends on executive functions. In this sample of healthy, high-performing adolescents, school performance may be affected more strongly by other cognitive factors, for example, content knowledge of the school subjects, or psychological factors such as motivation or personality. Moreover, the study illustrates that controlling for confounders is very important in research on the effect of executive functions on school performance (Willoughby et al., 2012). Future research may investigate whether these results also hold for other executive function tests and other samples. For instance, are similar results seen in adolescents who study at other educational levels? And in adolescents who repeated a grade or have a developmental disorder? Based on the current study, we can conclude that the executive functions measured with the Sorting Test, Tower Test, and BRIEF-SR do not play a major role in report marks obtained by healthy, high-performing adolescents.

## Author contributions

All authors contributed to the design and execution of the study. All authors drafted and revised the study, and approved the final version. All authors agreed to be accountable for all aspects of the work.

### Conflict of interest statement

The authors declare that the research was conducted in the absence of any commercial or financial relationships that could be construed as a potential conflict of interest.
